# A Procedure for Precise Determination and Compensation of Lead-Wire Resistance of a Two-Wire Resistance Temperature Detector

**DOI:** 10.3390/s22114176

**Published:** 2022-05-31

**Authors:** Apinai Rerkratn, Supatsorn Prombut, Thawatchai Kamsri, Vanchai Riewruja, Wandee Petchmaneelumka

**Affiliations:** 1 School of Engineering, King Mongkut’s Institute of Technology Ladkrabang, Bangkok 10520, Thailand; apinai.re@kmitl.ac.th (A.R.); 63601068@kmitl.ac.th (S.P.); 2 Thai Microeletronics Center (TMEC), Chachoengsao 24000, Thailand; thawatchai.kamsri@nectec.or.th

**Keywords:** resistance temperature detector, lead-wire resistance, lead-wire compensation, remote measurement, three-level pulse signal, voltage-to-current converter

## Abstract

A procedure for the precise determination and compensation of the lead-wire resistance of a resistance transducer is presented. The proposed technique is suitable for a two-wire resistance transducer, especially the resistance temperature detector (RTD). The proposed procedure provides a technique to compensate for the lead-wire resistance using a three-level pulse signal to excite the RTD via the long lead wire. In addition, the variation in the lead-wire resistance disturbed by the change in the ambient temperature can also be compensated by using the proposed technique. The determination of the lead-wire resistance from the proposed procedure requires a simple computation method performed by a digital signal processing unit. Therefore, the calculation of the RTD resistance and the lead-wire resistance can be achieved without the requirement of a high-speed digital signal processing unit. The proposed procedure is implemented on two platforms to confirm its effectiveness: the LabVIEW computer program and the microcontroller board. Experimental results show that the RTD resistance was accurately acquired, where the measured temperature varied from 0 °C to 300 °C and the lead-wire resistance varied from 0.2 Ω to 20 Ω, corresponding to the length of the 26 American wire gauge (AWG) lead wire from 1.5 m to 150 m. The average power dissipation to the RTD was very low and the self-heating of the RTD was minimized. The measurement error of the RTD resistance observed for pt100 was within ±0.98 Ω or ±0.27 °C when the lead wire of 30 m was placed in an environment with the ambient temperature varying from 30 °C to 70 °C. It is evident that the proposed procedure provided a performance that agreed with the theoretical expectation.

## 1. Introduction

Temperature is the most monitored and controlled variable in industrial control systems. A temperature sensor plays the important role in industrial applications of maintaining a certain temperature for a specific process. There are several common types of temperature sensors used in industrial process applications, such as a negative coefficient thermistor (NTC), resistance temperature detector (RTD), thermocouple, and semiconductor junction-based sensor [1,2,3]. The NTC, thermocouple, and semiconductor junction-based sensor require a complex signal conditioning circuit to measure the temperature. The RTD is a passive sensor and produces a positive variation in its resistance to the variation of ambient temperature. The RTD is constructed from platinum wire, which provides distinctive behaviors in terms of high accuracy, high linearity, high stability, and a low hysteresis effect [4,5,6]. In industrial applications, the RTD has become the most considered sensor for temperature measurement due to its behaviors. The distance from the RTD sensor to the signal conditioning circuit requires a long-range lead wire, which is considered as the parasitic resistance for each lead wire. The lead-wire resistance of the wire connected from the RTD to the control station includes the RTD resistance. This is introduced as a large error of temperature measurement in the industrial process. Moreover, the change in the ambient temperature causes variation in the lead-wire resistance due to the thermal property of the metals used for the lead wire, which causes an uncertain error in the measured temperature quantity. If the lead-wire resistance of about 1 Ω is added to the RTD resistance, then the measured temperature error occurs at about 2.4 °C for a pt100 two-wire RTD [7,8,9,10,11]. In addition, the contact resistance of the terminal between the control station and the RTD is added to the lead-wire resistance. The contact resistance including the lead-wire resistance is collectively called the lead-wire resistance in this paper. A drawback of the RTD sensor is the lead-wire resistance, which can be compensated using the RTD with three or four lead wires [2,3,4,5,12,13]. The range of all the lead wires is set to equal, which provides equal lead-wire resistance. Usually, the determination of the process temperature is based on the use of a bridge circuit [1,2,3,11,13,14,15]. A disadvantage of the bridge circuit is that the signal readout directly from the bridge circuit is nonlinear. The technique for a linear readout of the signal from the bridge circuit using a microcontroller and relaxation oscillator is proposed in the literature [10]. The compensation of the lead-wire resistance using a three- or four-wire RTD is also proposed in the recent literature [12,16]. These techniques require several amplifiers and a current source, which adds complexity to the signal conditioning circuit. However, the compensation of lead-wire resistance for three- and four-wire RTDs is efficient only when there is equal resistance in each lead wire. In addition, the RTD with three or four lead wires increases the cost and complexity of the control system, especially for the remote measurement and the multipoint measurements. Therefore, the use of a two-wire RTD sensor is a simple and economical attraction. There are many techniques to reduce the error due to the lead-wire resistance, including the contact resistance of the two-wire resistive sensor, proposed in the literature [7,8,9,10,13,17]. An approach to compensate for the error caused by the lead-wire in a two-wire RTD using a precision shunt voltage reference and current source is proposed in [18]. This technique provides two kinds of excitation signals, a voltage signal and a current signal, for the RTD. The voltage signal from the precision voltage reference of this approach is used to measure the lead-wire resistance, whereas the current signal from the current source is used to obtain the resistance of the RTD, including the lead-wire resistance. Unfortunately, the high magnitude of the voltage signal across the RTD during the lead-wire resistance measurement causes a self-heating effect in the RTD. Therefore, this approach is suitable for when the RTD has a high resistance, such as the pt1000 RTD has, to reduce the current flowing through the RTD. Other approaches based on two diodes acting as an electronic switch connected to the terminal of the RTD to measure the lead-wire resistance are proposed in the recent literature [7,8,9,10,18]. The operation of these approaches is based on the relaxation oscillator and microcontroller. These approaches require the bidirectional current signal to obtain the resistance of the RTD. However, the threshold voltage of the diodes must be equal to avoid the error of the measured variable. For the mismatch of a diode threshold voltage of about 1 mV, the measured error of about 2.5 °C was observed for the pt100 RTD with a 1 mA excitation current [11]. Furthermore, the diode threshold voltage is dependent on the variation in the ambient temperature due to the thermal voltage that is characteristic of diodes. This error becomes a significant parameter, same as the lead-wire resistance. To avoid the error mentioned above, closely matched diodes are required. Unfortunately, the closely matched diodes are impossible for discrete devices. The technique using two diodes is low cost and simple without using specific devices. If the threshold voltage of the diode can be compensated for, then the advantage of this technique will be gained.

In this paper, the procedure to determine and compensate for the lead-wire resistance and the resistance of the RTD using two diodes is proposed. The lead-wire resistance, the RTD resistance, and the diode threshold voltage are accurately determined. The effect of the mismatch of the diodes in terms of the threshold voltage can be prevented with the proposed technique. The proposed procedure provides the bidirectional current signal with three-step pulse levels in the amplitude of the series to excite the RTD sensor. The amplitude of each step of the current signal is doubled in the amplitude of the series. The RTD resistance, the lead-wire resistance, and the diode threshold voltage are determined via a digital signal processing unit using the simple mathematic operations add and subtract. Therefore, the operation time of the proposed procedure is fast and accurate. The proposed procedure is implemented to confirm the accuracy and performance using commercial devices. The temperature readout from the proposed technique exhibits that a maximum error of about 0.27 °C is observed, where the ambient temperature varies from 30 °C to 70 °C. It should be noted that the proposed technique can be applied to many types of resistive sensors in related fields such as civil engineering [19,20,21,22,23,24], automobile engineering [25,26], and mechanical engineering [27], and in the use of scientific and medical equipment [3,4,5,6,28], to measure the quantity of gas, humidity, airflow, force, pressure, and strain. For the example of the application in the field of civil engineering, the resistive sensors are provided for the measurement of the structural parameters to evaluate the structural behaviors of the structural health monitoring system [19,20,21,22,23,24]. In addition, the proposed technique can be applied to investigate the behaviors of construction materials such as fly-ash-based concrete and non-autoclaved silicate materials [29,30]. The resistance readout from the resistive sensor requires an accurate value to make a decision regarding the safety of civil infrastructure [20,24]. Therefore, the proposed technique is suitable for this requirement. The organization of this paper is divided into five sections as follows: Section 2 introduces a conventional technique to compensate for the lead-wire resistance. The proposed procedure and circuit to determine the resistance of the lead wire and the RTD using a three-step pulse signal are also presented in this section. In Section 3, the accuracy of the proposed circuit is analyzed and discussed in detail. Section 4 describes the experimental results of the proposed procedure using two signal processing units. The first signal processing unit is the LabVIEW computer-based program interfaced with an analog input/output board. Another signal processing unit is a microcontroller board. Finally, the conclusion of this paper is described in Section 5.

## 2. Principle of the Proposed Procedure

The accuracy of the temperature measurement using the RTD is disturbed by the parasitic resistance of the lead wire. The resistance readout from the RTD includes the lead-wire resistance. In addition, the lead-wire resistance is dependent on the change in the ambient temperature, which can cause an uncertain readout of the resistance from the RTD. The compensation of the lead-wire resistance using two diodes is a useful technique and the procedure for determining the lead-wire resistance is described in this section.

### 2.1. Conventional Procedure 

**Figure 1:**
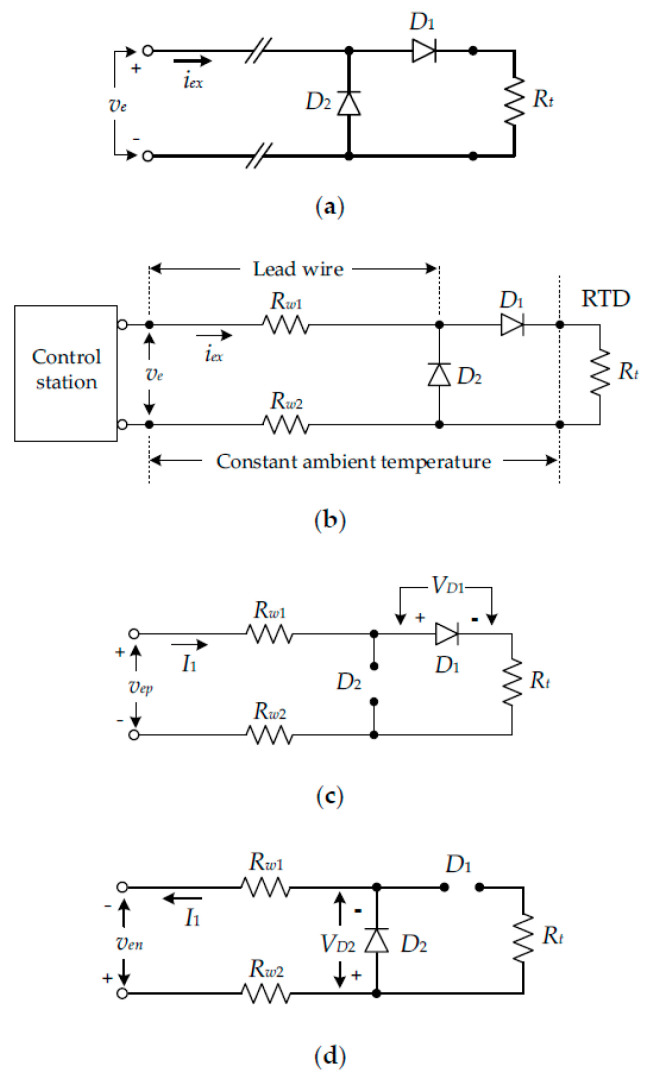
Lead-wire resistance compensation technique using two diodes: (**a**) simple diagram; (**b**) equivalent diagram; (**c**) equivalent diagram for *i_ex_* = *I*_1_; (**d**) equivalent diagram *i_ex_* = −*I*_1_.

A diagram of the RTD connected with two diodes is shown in Figure 1a, where *R_t_* is the RTD resistance [7,8,9,10,17]. Two diodes are laid close to the terminals of the RTD to ignore the wiring resistance between the RTD resistance *R_t_* and the diodes. The lead-wire resistance is determined from the RTD to the control station as shown in Figure 1b, where *R_w_*_1_ and *R_w_*_2_ are the intrinsic resistances of two lead wires. For recent approaches, the voltages across the diodes *D*_1_ and *D*_2_ were assumed to be equal. The lead-wire resistance was compensated for in these approaches using bidirectional excitation current *i_ex_*. From Figure 1c, the excitation current *i_ex_* = *I*_1_ is applied; then, the diodes *D*_1_ and *D*_2_ are conducted and turned off, respectively, as shown in Figure 1c. The relationship between the voltage signal *v_ep_* and the excitation current *i_ex_* can be given by:

(1) $$v_{ep}=I_{1}R_{t}+I_{1}R_{w1}+I_{1}R_{w2}+V_{D1}$$

where *V_D_*_1_ is the voltage across the diode *D*_1_. For the excitation current *i_ex_* = −*I*_1_, the operations of the diodes *D*_1_ and *D*_2_ are opposite from the previous state, as shown in Figure 1d. Therefore, the voltage *v_en_* can be stated as:

(2) $$v_{en}=I_{1}R_{w1}+I_{1}R_{w2}+V_{D2}$$

where *V_D_*_2_ is the voltage across the diode *D*_2_. If the closely matched diodes are chosen for diodes *D*_1_ and *D*_2_, then *V_D_*_1_ = *V_D_*_2_ is obtained. Practically, the intrinsic resistances *R_w_*_1_ and *R_w_*_2_ are equal due to the same length of the two lead wires. Therefore, the RTD resistance *R_t_* can be simply obtained by the subtraction of Equations (1) and (2) as:

(3) $$R_{t}=\frac{\left(v_{ep}-v_{en}\right)}{I_{1}}$$

It should be noted that the RTD resistance of Equation (3) is accurately determined only for the constant ambient temperature of the lead wire and the diodes in Figure 1b. In addition, the mismatch of the diodes causes the incomplete cancellation of the voltage across the diodes *D*_1_ and *D*_2_. The voltage *V_D_* across the diode is dependent on the ambient temperature and can be expressed as:

(4) $$V_{D}=\eta \frac{kT}{q}\ln\frac{I_{D}}{I_{S}}$$

where *η* and *I_S_* are the empirical constant and the reverse saturated current of the diode, respectively, *k* = 1.38 × 10^−^^23^ J/K is the Boltzmann constant, *q* = 1.602 × 10^−^^19^ C is the electron charge, and *T* = (273 + °C) is the absolute temperature in Kelvin. It should be noted that the term *kT*/*q* is usually called the thermal voltage *V_T_* [31]. The thermal voltage *V_T_* is approximated as 25.67 mV at 25 °C of the ambient temperature. In Equation (4), the mismatched diodes are exhibited in terms of the reverse saturated current *I_S_* of each diode. Practically, the perfectly matched diodes are not enough in the discrete device and also in the integrated circuit form. In Equation (3), the incomplete cancellation of the diode voltages *V_D_*_1_ and *V_D_*_2_ causes the calculation error of the resistance *R_t_*. The voltage across the diode can be accurately determined using the proposed procedure. Therefore, the requirement of perfectly matched diodes is unnecessary.

### 2.2. Proposed Procedure

**Figure 2:**
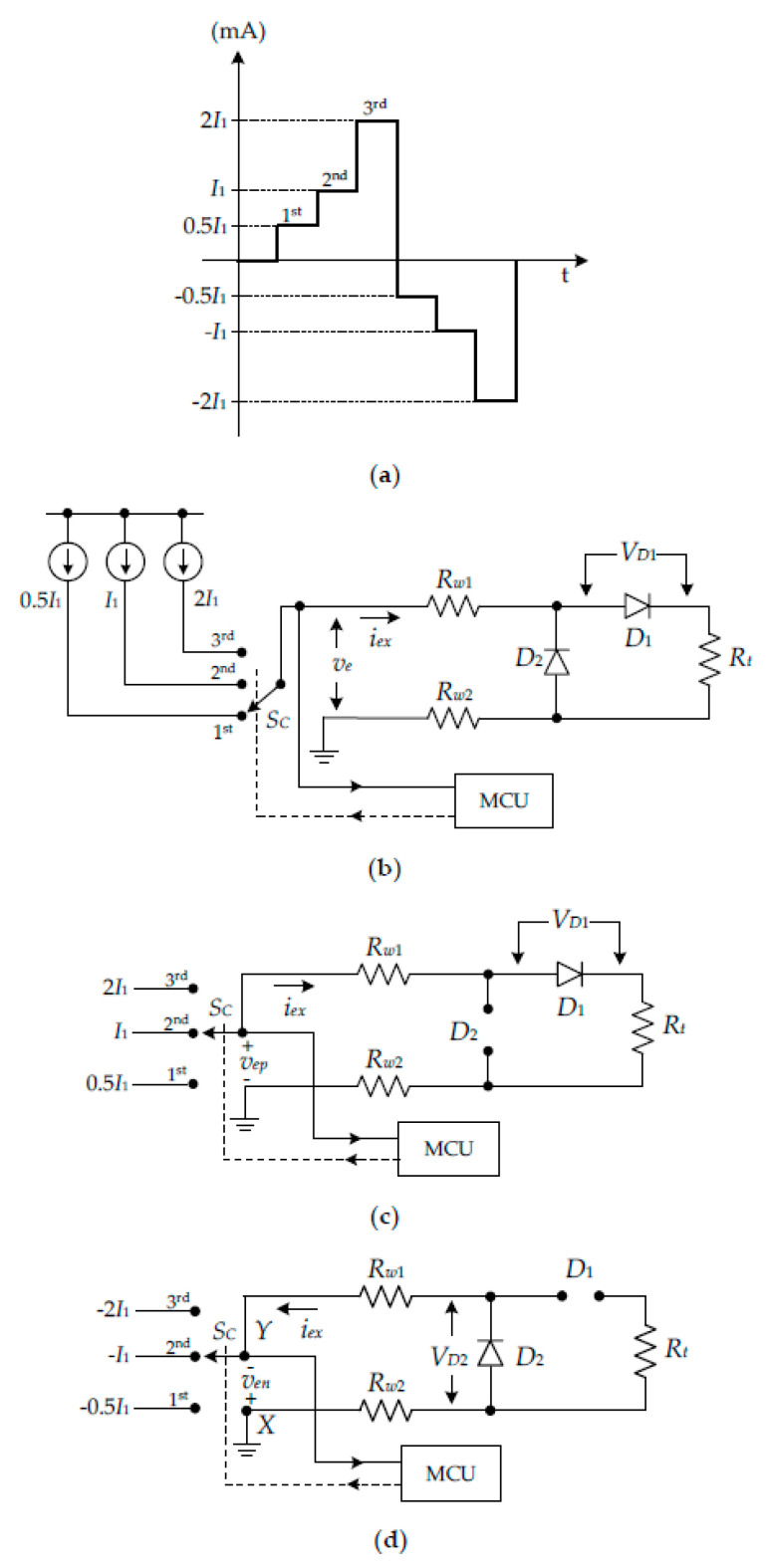
Proposed procedure for determination of lead-wire resistance and diode voltages: (**a**) three-step current signal; (**b**) operation diagram of proposed procedure; (**c**) operation diagram for stage I; (**d**) operation diagram for stage II.

To determine the voltage across diodes *V_D_*_1_ and *V_D_*_2_, the current signal in the form of a three-step pulse signal is provided to excite the RTD as shown in Figure 2a. Each step of the current signal is double the previous current quantity. All resistances in the current signal path can also be simply determined. The circuit diagram for the operation of the proposed procedure is shown in Figure 2b. In Figure 2b, the three-step pulse signal is simplified by three current sources for the explanation of the circuit operation. The microprocessor and controller unit (MCU) is used for the digital signal processing and controlling the analog switch *S_C_* to generate the three-step pulse signal. The operation of the circuit diagram in Figure 2b can be considered in two stages of the excitation signals: stage I for the positive current signal and stage II for the negative current signal. For stage I of the procedure, the magnitude *I*_1_ of the excitation current *i_ex_* in the second step in Figure 2a is set as the reference current. The current magnitudes of the first step and third step are assigned to equal *I*_1_/2 and 2*I*_1_, respectively. An operating diagram for stage I is shown in Figure 2c. In Figure 2c, the diodes *D*_1_ and *D*_2_ are conduct and cutoff, respectively. For the first step of the excitation current *i_ex_* = *I*_1_/2, the voltage *v_ep_*_1_ can be stated as:

(5) $$v_{ep1}=\left(R_{w1}+R_{w2}+R_{t}\right)\frac{I_{1}}{2}+V_{D1}=\left(R_{w1}+R_{w2}+R_{t}\right)\frac{I_{1}}{2}+\eta V_{T}\ln\frac{I_{1}}{2I_{S1}}$$

where *I_S_*_1_ denotes the reverse saturated current of the diode *D*_1_. The voltage *v_ep_*_1_ is converted to digital form by an analog-to-digital converter (ADC) and transferred to the MCU. For the second step of the excitation current *i_ex_* = *I*_1_, the voltage *v_ep_*_2_ can be given by:

(6) $$v_{ep2}=\left(R_{w1}+R_{w2}+R_{t}\right)I_{1}+\eta V_{T}\ln\frac{I_{1}}{I_{S1}}$$

The voltage *v_ep_*_22_ is assigned as double the magnitude of the voltage *v_ep_*_2_, which is calculated by the MCU. Therefore, the voltage *v_ep_*_22_ can be written as:

(7) $$v_{ep22}=2\left(R_{w1}+R_{w2}+R_{t}\right)I_{1}+2\eta V_{T}\ln\left(\frac{I_{1}}{I_{S1}}\right)$$

It should be noted that doubling the voltage across diode *V_D_*_1_, written as 2*V_D_*_1_, corresponds to the term of *ηV_T_*ln(*I*_1_/*I_S_*_1_)^2^. Therefore, Equation (7) can be rewritten as:

(8) $$v_{ep22}=2\left(R_{w1}+R_{w2}+R_{t}\right)I_{1}+\eta V_{T}\ln{\left(\frac{I_{1}}{I_{S1}}\right)}^{2}$$

For the third step, the excitation current *i_ex_* = 2*I*_1_ is set. Then, the voltage *v_ep_*_3_ can be expressed as:

(9) $$v_{ep3}=2\left(R_{w1}+R_{w2}+R_{t}\right)I_{1}+\eta V_{T}\ln\frac{2I_{1}}{I_{S1}}$$

From Equations (8) and (9), the subtraction result *v_eps_*_1_ of the voltages *v_ep_*_22_ and *v_ep_*_3_ can be given by:

(10) $$v_{eps1}=\eta V_{T}\left(\ln{\left(\frac{I_{1}}{I_{S1}}\right)}^{2}-\ln\left(\frac{2I_{1}}{I_{S1}}\right)\right)=\eta V_{T}\ln\frac{I_{1}}{2I_{S1}}$$

To subtract Equation (5) by Equation (10), the resulting voltage *v_eps_*_2_ can be given as:

(11) $$v_{eps2}=\frac{I_{1}}{2}R_{t}+\frac{I_{1}}{2}\left(R_{w1}+R_{w2}\right)$$

Equations (10) and (11) exhibit only the voltage across diode *V_D_*_1_ and all resistances in the current path, respectively. Practically, the resistances *R_w_*_1_ = *R_w_*_2_ = *R_w_* are assigned due to the same length of the lead wire. Therefore, Equation (11) can be rewritten as:

(12) $$v_{eps2}=\frac{I_{1}}{2}R_{t}+I_{1}R_{w}$$

The resistance *R_w_* can be obtained by stage II of the proposed procedure. The operating circuit for stage II is shown in Figure 2d. The magnitude of the excitation current *i_ex_* for stage II is set as a negative current. Therefore, the magnitudes of the excitation currents of each step are set as –*I*_1_/2, −*I*_1_, and −2*I*_1_. From Figure 2d, the excitation current *i_ex_* = *−I*_1_/2 is applied, which forces the diodes *D*_1_ and *D*_2_ to cutoff and conduct, respectively. Therefore, the voltage *v_en_*_1_ across nodes X and Y can be given by:

(13) $$v_{en1}=I_{1}R_{w}+\eta V_{T}\ln\left(\frac{I_{1}}{2I_{S2}}\right)$$

where *I_S_*_2_ is the reverse saturated current of the diode *D*_2_. As the same with stage I, the voltage *v_en_*_2_ can be expressed for the excitation current *i_ex_* = −*I*_1_ in the second step as:

(14) $$v_{en2}=2I_{1}R_{w}+\eta V_{T}\ln\left(\frac{I_{1}}{I_{S2}}\right)$$

The voltage *v_en_*_2_ in Equation (14) is multiplied by two as:

(15) $$v_{en22}=2v_{en2}=4I_{1}R_{w}+\eta V_{T}\ln{\left(\frac{I_{1}}{I_{S2}}\right)}^{2}$$

For the excitation current *i_ex_* = −2*I*_1_ in the third step, the voltage *v_en_*_3_ across nodes X and Y can be stated as:

(16) $$v_{en3}=4I_{1}R_{w}+\eta V_{T}\ln\left(\frac{2I_{1}}{I_{S2}}\right)$$

The subtraction result *v_ens_*_1_ of the Equations (15) and (16) can be given by:

(17) $$v_{ens1}=\eta V_{T}\left(\ln{\left(\frac{I_{1}}{I_{S2}}\right)}^{2}-\ln\left(\frac{2I_{1}}{I_{S2}}\right)\right)=\eta V_{T}\ln\left(\frac{I_{1}}{2I_{S2}}\right)$$

From Equation (17) the voltage *V_D_*_2_ across diode *D*_2_ is obtained. The diode voltage *V_D_*_2_ is subtracted from Equation (13) as:

(18) $$v_{ens2}=v_{en1}-v_{ens1}=I_{1}R_{w}$$

From Equation (18), the lead-wire resistance *R_w_* = *v_ens_*_2_/*I*_1_ is obtained. It should be noted that the RTD resistance *R_t_* can be achieved by substituting Equation (18) in Equation (12) as:

(19) $$R_{t}=\frac{2\left(v_{eps2}-v_{ens2}\right)}{I_{1}}$$

The advantage of the proposed procedure is that the RTD resistance *R_t_*, the resistances *R_w_*_1_ = *R_w_*_2_, and the diode voltages *V_D_*_1_ and *V_D_*_2_ can be accurately determined.

### 2.3. Implementation of the Proposed Procedure

**Figure 3:**
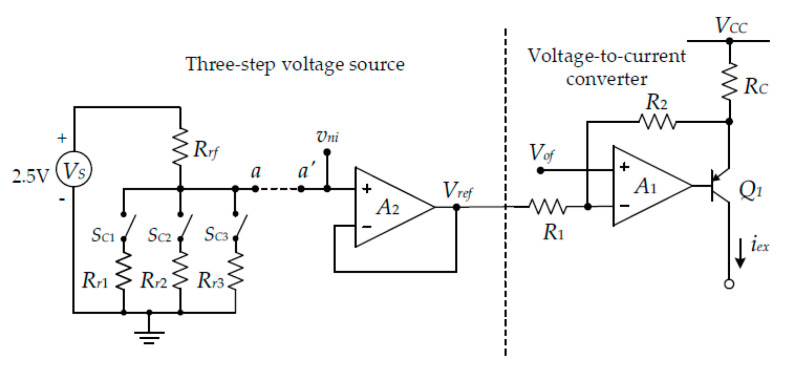
Three-step current source.

The proposed procedure is realized using a mixed-signal circuit technique, which contains both analog and digital properties. The three-step pulse signal of Figure 2a can be realized as shown in Figure 3, which is separated into two parts, a three-step voltage source and a voltage-to-current converter. The voltage-to-current converter, shown on the right side of the circuit in Figure 3, consists of an operational amplifier (opamp) *A*_1_, transistor *Q*_1_, and resistors *R*_1_, *R*_2_, and *R_C_*. From the routine circuit analysis, the relationship between the excitation current *i_ex_* and the voltage *V_ref_* can be expressed as:

(20) $$i_{ex}=\frac{R_{2}}{R_{1}R_{C}}V_{ref}+\frac{V_{CC}}{R_{C}}-\frac{\left(R_{1}+R_{2}\right)}{R_{1}R_{C}}V_{of}$$

where *V_CC_* is the power-supply voltage of the opamp *A*_1_. From Equation (20), if the conditions of *R*_1_ = *R*_2_, *V_of_* = *V_CC_*/2, and *R*_2_ >> *R_C_* are fulfilled, then the excitation current *i_ex_* can be stated as:

(21) $$i_{ex}=\frac{V_{ref}}{R_{C}}$$

From Equation (21), the current quantity of each step of the excitation current *i_ex_* can be obtained by changing the reference voltage *V_ref_* to an appropriate value. The reference voltage *V_ref_* in Equation (21) is provided from the three-step voltage source on the left side of the circuit in Figure 3. 

The analog switches *S_C_*_1_, *S_C_*_2_, and *S_C_*_3_, and the resistances *R_r_*_1_, *R_r_*_2_, and *R_r_*_3_ are one-by-one controlled by the MCU to achieve the currents for each step. The voltage source *V_S_* provides the constant voltage to generate the reference voltage *V_ref_*. The opamp *A*_2_ acts as the voltage follower used to prevent the loading effect. For the proposed procedure, the resistances *R_r_*_1_ to *R_r_*_3_ are successively connected by means of the MCU-controlled analog switches *S_C_*_1_, *S_C_*_2_, and *S_C_*_3_ to provide the magnitude of each step for the excitation current *i_ex_* as *I*_1_/2, *I*_1_, and 2*I*_1_. The reference voltage *V_ref_* can be simply calculated as:

(22) $$V_{refi}=\frac{R_{ri}V_{S}}{\left(R_{rf}+R_{ri}\right)}\;\;\mathrm{for}i=1,\;2,\;3$$

The voltage *V_ref_* is applied to the voltage-to-current converter to generate the three-step current signal. Therefore, the excitation currents *i_ex_* of each step, *I*_1_/2, *I*_1_, and 2*I*_1_, are obtained.

**Figure 4:**
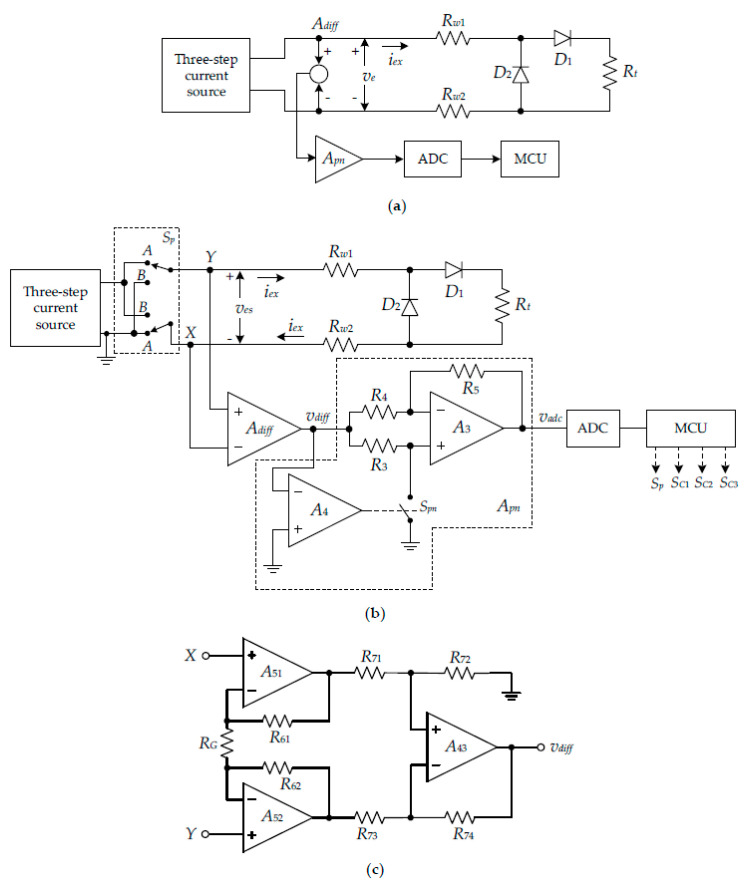
Proposed technique to acquire the RTD resistance: (**a**) block diagram of proposed technique; (**b**) circuit diagram of proposed technique; (**c**) schematic diagram of *A_diff_*.

The block diagram of the proposed circuit technique is shown in Figure 4a. As seen in Figure 4a, the analog signal circuit consists of the three-step current source, the difference amplifier *A_diff_*, and the phase-inversion-switched amplifier *A_pn_* [32]. 

The digital signal circuit comprises the analog-to-digital converter (ADC) and the MCU, where both ADC and MCU are integrated into the microcontroller board for practical implementation. The simplified diagram of the proposed procedure is shown in Figure 4b. In Figure 4b, the set of an analog switch unit *S_P_* is used to control the flow direction of the excitation current *i_ex_*. The operation of the diagram in Figure 4b can be explained as follows. For stage I of the proposed procedure, the MCU controls the switch unit *S_P_* and sets to the position “A”. The excitation current *i_ex_* flows through the resistance *R_w_*_1_, the diode *D*_1_, the resistance *R_t_*, and the resistance *R_w_*_2_. In Figure 4b, the analog voltage *v_es_* is amplified to a proper value by the difference amplifier *A_diff_*. The schematic diagram of the difference amplifier *A_diff_* is shown in Figure 4c. From Figure 4c, the resistances *R*_61_ = *R*_62_ = *R*_6_ and *R*_71_ = *R*_72_ = *R*_73_ = *R*_74_ = *R*_7_ are assigned. Then, output signal *v_diff_* of the difference amplifier *A_diff_* can be expressed as [28,33]:

(23) $$v_{diff}=\left(1+\frac{2R_{6}}{R_{G}}\right)v_{es}=G_{a}v_{es}$$

The phase-inversion-switched amplifier *A_pn_*, consisting of the opamps *A*_3_ and *A*_4_, the resistors *R*_3_ to *R*_5_, and analog switch *S_pn_*, is provided to invert the negative voltage signal *v_diff_* of the second stage of the proposed procedure to the positive voltage signal for the ADC. The opamp *A*_4_ acts as a comparator to investigate the polarity of the voltage signal *v_es_*. If the voltage signal *v_es_* is positive for stage I and the switch *S_pn_* is controlled to “open” by the opamp *A*_4_, then the phase-inversion-switched amplifier *A_pn_* is formed as the noninverting amplifier with unity gain. For stage II, the voltage signal *v_es_* is negative and the switch *S_pn_* is controlled to “close” by the opamp *A*_4_. Therefore, the phase-inversion-switched amplifier *A_pn_* acts as the inverting amplifier with unity gain. The transfer characteristic of the phase-inversion-switched amplifier *A_pn_* can be expressed as:

(24) $$v_{adc}=\{\begin{array}{c} G_{a}v_{esi}\\ -G_{a}v_{esi} \end{array}\;\;\begin{array}{c} \;for\;v_{esi}>0\\ \;for\;v_{esi}<0 \end{array}$$

where *i* = 1, 2, 3 for the signal currents *I*_1_/2, *I*_1_, and 2*I*_1_. The voltage signals *v_es_*_1_, *v_es_*_2_, and *v_es_*_3_ of each step are successively obtained and transferred to the MCU. In the same way, the analog switch unit *S_P_* is controlled by MCU to position “B”, which causes the excitation current *i_ex_* to flow in the opposite direction. The excitation current *i_ex_* flows through the resistance *R_w_*_2_, the diode *D*_2_, and the resistance *R_w_*_1_. Each step of the voltage signals *v_es_*_1_ to *v_es_*_3_ is sequentially transferred to the MCU. Therefore, the resistances *R_w_*_1_, *R_w_*_2_, and the voltage across the diode *D*_2_ in the excitation current path are determined by the proposed procedure mentioned in Section 2.2.

## 3. Performance Analysis

The accuracy of the proposed technique to determine the lead-wire resistance, the RTD resistance, and the voltage across the diode can be disturbed by the nonideal characteristic of the devices used in the experimental circuit. There are three major factors that cause an error in the proposed technique. The first factor, the derivation from the expected magnitude of the excitation current *i_ex_* for each step in stage I and stage II, causes an inaccuracy of the calculated resistances and diode voltages in the proposed procedure. For the voltage-to-current converter circuit in Figure 3, the tolerance of the resistors used in the experimental circuit causes an error on the excitation current *i_ex_*. From Equation (20), the relationship between the current *i_ex_* and the reference voltage source *V_ref_* including the tolerance of the resistors can be approximately given by:

(25) $$i_{ex}=\left(1-\varepsilon_{i}\right)\frac{V_{ref}}{R_{C}}+I_{offset}$$

(26) $$\varepsilon_{i}=\frac{\Delta_{R}}{\left(1+\frac{\Delta_{R}}{2}\right)}$$

(27) $$I_{offset}=\frac{V_{CC}}{R_{C}}-\frac{\left(2+\Delta_{R}\right)}{\left(1+\frac{\Delta_{R}}{2}\right)}\frac{V_{of}}{R_{C}}$$

where Δ*_R_* denotes the tolerance of the resistor. From Equation (25), the tolerance Δ*_R_* of the resistors causes the error *ε_i_* on the converted current *i_ex_* in the first term on the right. This error can be avoided by replacing the resistor *R*_1_ in Figure 3 with the variable resistor and fine-tuning the resistance to match the resistance *R*_2_. In addition, the offset current *I_offset_* in Equation (27) can be canceled by adjusting the voltage *V_of_* to an appropriate value. The second factor, the gain error of the phase-inversion-switched amplifier in Figure 4c causes the error in the voltage signal *v_adc_*. The voltage signal *v_adc_*, from the phase-inversion-switched amplifier for the resistors *R*_3_ and *R*_4_ having the tolerance Δ*_P_*, can be approximated as:

(28) $$v_{adc}=\{\begin{array}{c} \frac{\left(1+\frac{3\Delta_{P}}{2}\right)}{\left(1+\frac{\Delta_{P}}{2}\right)}G_{a}v_{es}\;\;\;\;for\;v_{es}>0\\ \frac{-\left(1-\frac{\Delta_{P}}{2}\right)}{\left(1+\frac{\Delta_{P}}{2}\right)}G_{a}v_{es}\;\;\;\;for\;v_{es}<0\; \end{array}$$

In Equation (28), the closely matched selection of the resistors *R*_3_ and *R*_4_ can minimize the gain error of the phase-inversion-switched amplifier. The third factor is when the gain error occurs due to the mismatched resistors in the schematic diagram of the difference amplifier *A_diff_*. However, the difference amplifier *A_diff_* used for the experiment in this paper is commercially available, and the resistances in the schematic circuit are trimmed by laser to ensure the closely matched resistors [33]. Therefore, the gain error is very small and too insignificant to disturb the performance of the experimental implementation in this paper. It should be noted that the contact resistance of the analog switch unit *S_P_* is unaffected by the signal *v_es_* transferred to the difference amplifier *A_diff_*.

## 4. Experimental Results

**Figure 5:**
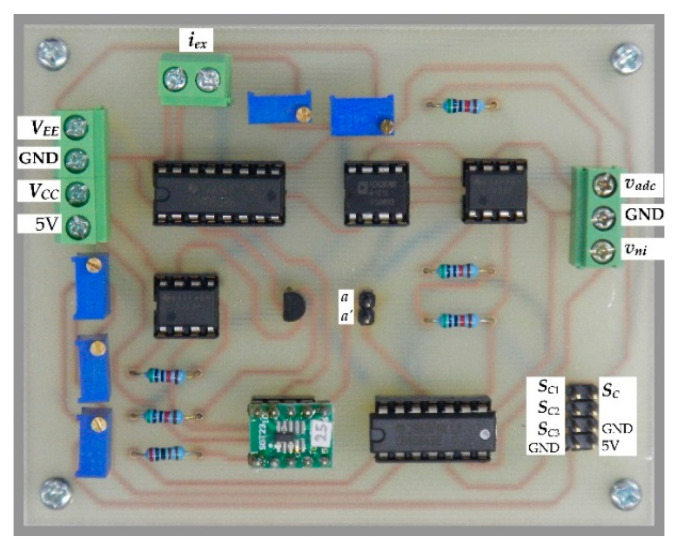
Prototype board of proposed circuit.

To confirm the effectiveness of the proposed procedure, the interfacing circuits in Figure 3 and Figure 4b were breadboarded. The active devices used in this experiment were the AD620 for difference amplifier *A_diff_*, LF353 for opamps *A*_1_, *A*_2_, *A*_3_, and *A*_4_, REF3025 for the constant voltage source *V_S_*, 2N3906 for PNP transistor *Q*_1_, 1N4148 for diodes *D*_1_ and *D*_2_, CD4066 for analog switches *S_C_*_1_ to *S_C_*_3_ and *S_pn_*, and CD4053 for analog switch unit *S_p_*. The constant voltage source *V_S_* provided the constant voltage of 2.5 V. The resistors *R*_1_ = *R*_2_ = *R*_3_ = *R*_4_ = *R*_5_ = 50 kΩ were assigned. The amplification factors *G_a_* for pt100 and pt1000 were set as 4 and 2, respectively. From the simplified schematic of AD620, the resistances *R*_61_ and *R*_62_ = 24.7 kΩ were provided [21]; therefore, the resistance *R_G_* for the amplification factors *G_a_* of 4 and 2 were calculated from Equation (23) as 8.23 kΩ and 49.4 kΩ, respectively. The variable resistor was used for the resistor *R_G_* to acquire the calculated resistance. The RTDs were pt100 and pt1000 with nominal resistances of 100 Ω and 1000 Ω, respectively, at 0 °C. The power supplies of the opamps and the MCU were *V_CC_* = −*V_EE_* = ± 15 V and *V_DD_* = 5 V, respectively. The voltage *V_of_* was set as 7.5 V. The prototype board of the practical circuit is shown in Figure 5.

It should be noted that the diodes *D*_1_ and *D*_2_ were placed at the same ambient temperature as the lead wire. The reverse saturated currents *I_S_*_1_ and *I_S_*_2_ were calculated from Equation (4) as 1.59 nA and 1.45 nA, respectively, where the voltage across diodes *V_D_*_1_ and *V_D_*_2_ were measured for the forward bias current *I_D_* of 1 mA. The empirical constant *η* =1.765 was achieved. The excitation current *I*_1_ was set as 1 mA. Therefore, each step of the three-step current *I*_1_/2, *I*_1_, and 2*I*_1_ was 0.5 mA, 1 mA, and 2 mA, respectively. The reference voltage *V_ref_* of each step was given as 0.5 V, 1 V, and 2 V. From Equation (22), the resistors *R_C_*, *R_rf_*, *R_r_*_1_, *R_r_*_2_, and *R_r_*_3_ were calculated as 1 kΩ, 2 kΩ, 8 kΩ, 3 kΩ, and 500 Ω, respectively. It should be noted that the variable resistors were provided for the resistors *R_r_*_1_ to *R_r_*_3_ and adjusted the resistance to meet the calculated values. The time period of each step for the excitation current of stage I and stage II was assigned as 10 ms. To avoid self-heating, the duty cycle of the excitation signal for RTD was assigned as 15%. Only the excitation current of stage I flowed through the RTD. The power dissipations in the pt100 and pt1000 RTDs were about 3.06 μW and 30.62 μW, respectively, which is very small. Therefore, the change of the RTD resistance due to self-heating can be neglected. There are two signal processing units provided for the verification of the proposed technique performance in this experiment. The first signal processing unit, the MCU in Figure 4b, was replaced by the LabVIEW computer-based measurement and control program (NI LabVIEW 2014) and interfaced with the analog input/output (AIO) board from National Instruments (NI-USB-6361). It should be noted that other programming languages, such as Python, C, and C++, can also be used instead of the LabVIEW program. However, the specific programming for interfacing between the computer and AIO board, screen display, and signal processing need to be developed, which is inconvenient to implement in this experiment. The LabVIEW exhibits the ability to control, interface, and display, which has fulfilled the objectives of many researchers [34,35,36]. The second signal processing unit, the microcontroller provided by the ARM cortex STM32 Nucleo-64 board and including the LCD display, was used for the MCU [37]. The RTD resistances *R_t_* for pt100 and pt1000 were simulated via the resistance decade box for the variation in temperature from 0 °C to 300 °C, corresponding to the resistance 100 Ω to 213.93 Ω and 1000 Ω to 2139.3 Ω, respectively. The length of the lead wire, made of 26AWG copper wire, was simulated by the resistance decade box to achieve the equivalent length [11]. In this paper, the lead-wire resistance varied from 0.2 Ω to 20 Ω for the equivalent length of 1.5 m to 150 m, respectively.

**Figure 6:**
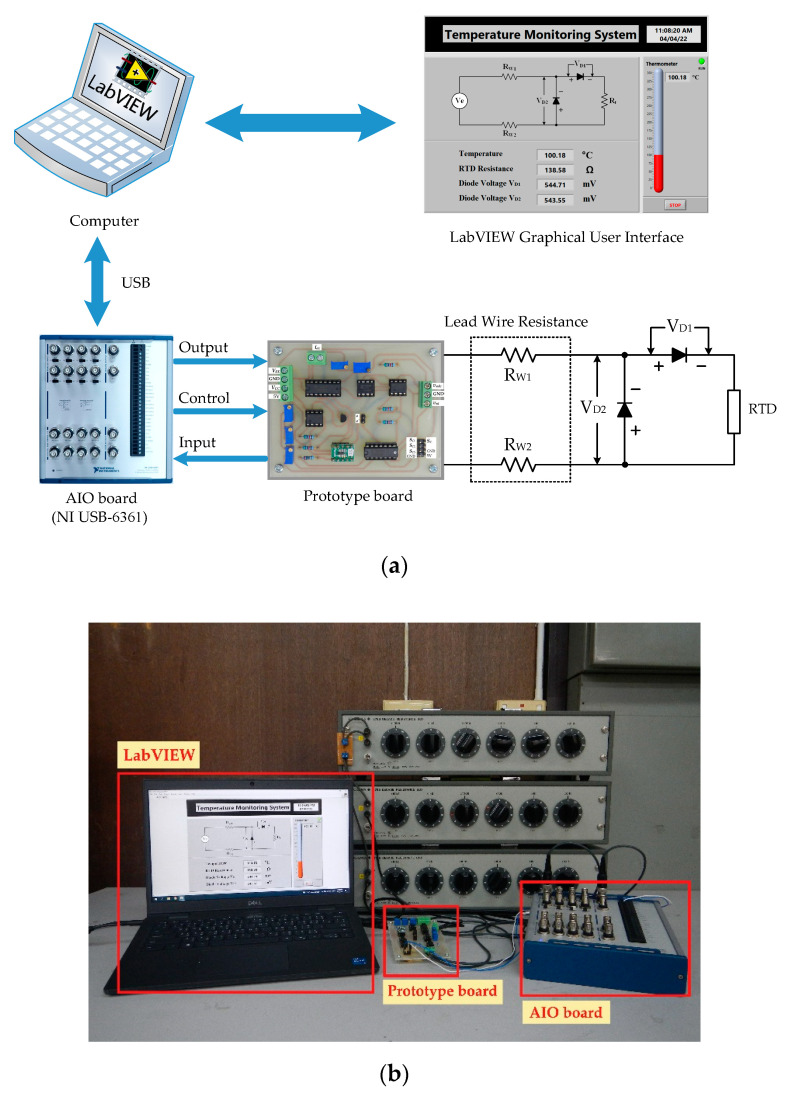

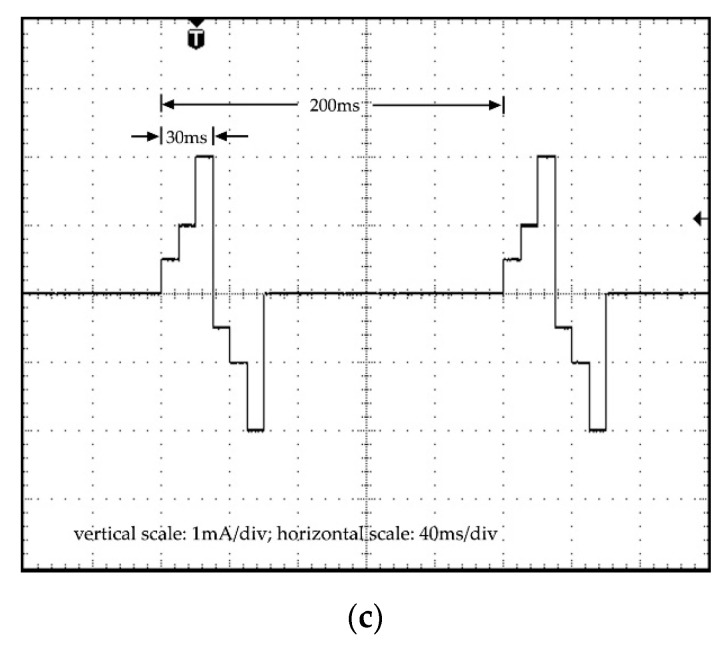
Experimental setup for MCU using LabVIEW: (**a**) block diagram; (**b**) experiment prototype; (**c**) waveform of three-step pulse signal *v_ni_*.

**Figure 7:**
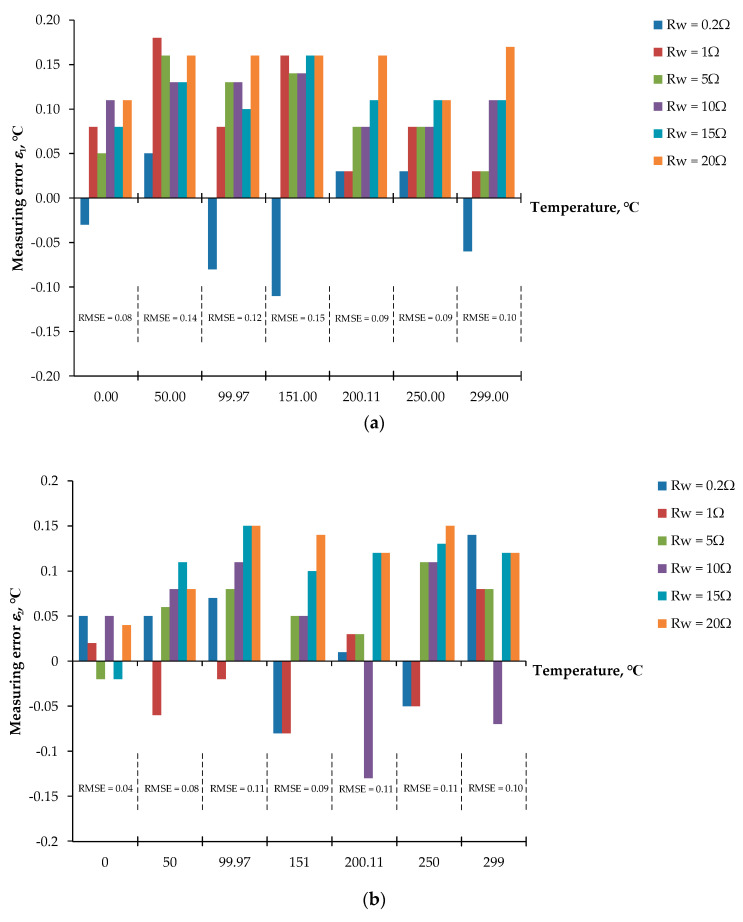
Errors of temperature measurement using LabVIEW and AIO board: (**a**) temperature error for pt100; (**b**) temperature error for pt1000.

For the first signal processing unit, Equations (5) to (10) and Equations (11) to (16) for stage I and stage II, respectively, of the proposed procedure including the three-step pulse signal were established by the LabVIEW program. The AIO board was provided to interface with the LabVIEW program to acquire the voltage signal *v_adc_* from the prototype board and, simultaneously, control the prototype board to generate the three-step current signal *i_ex_* to excite the RTD. Figure 6a,b show the connection diagram and the experimental setup of the proposed technique based on the LabVIEW program, respectively. From Figure 3, the excitation signal *i_ex_* was generated by removing the dashed line *a* and *a*’; then, the three-step pulse signal established from the LabVIEW program interfaced with the AIO board was applied as a voltage signal *v_ni_*. The bidirectional current signal was controlled by the switch *S_p_* and commanded from the LabVIEW program. The waveform of the three-step current signal *i_ex_* generated from the prototype board is shown in Figure 6c. The determinations of the RTD resistance *R_t_* and the diode voltages *V_D_*_1_ and *V_D_*_2_ are exhibited in Table 1, where the pt100 RTD is provided for this implementation. The error *ε*_1_ of the measured temperature from the RTD is shown in Figure 7a. The maximum error of the measured temperature of about 0.18 °C was observed. As seen in Figure 7a, the root mean square error (RMSE) for the dataset of the measured error *ε*_1_ was in the range of 0.08 °C to 0.15 °C, respectively. Subsequently, Table 2 and Figure 7b show the measured values and the error *ε*_2_ of the measured temperature, respectively, for pt1000 with the same condition as the previous experiment. As seen in Figure 7b, the maximum error was about 0.15 °C and the RMSE varied in the range of 0.04 °C to 0.11 °C. It should be noted that the error *ε*_1_ is slightly higher than error *ε*_2_ due to the sensitivity of the pt1000 being higher than that of th pt100. In addition, both errors *ε*_1_ and *ε*_2_ were caused by the residue error of the determination for the reverse saturated currents *I_S_*_1_ and *I_S_*_2_ of the diodes *D*_1_ and *D*_2_, respectively.

**Figure 8:**
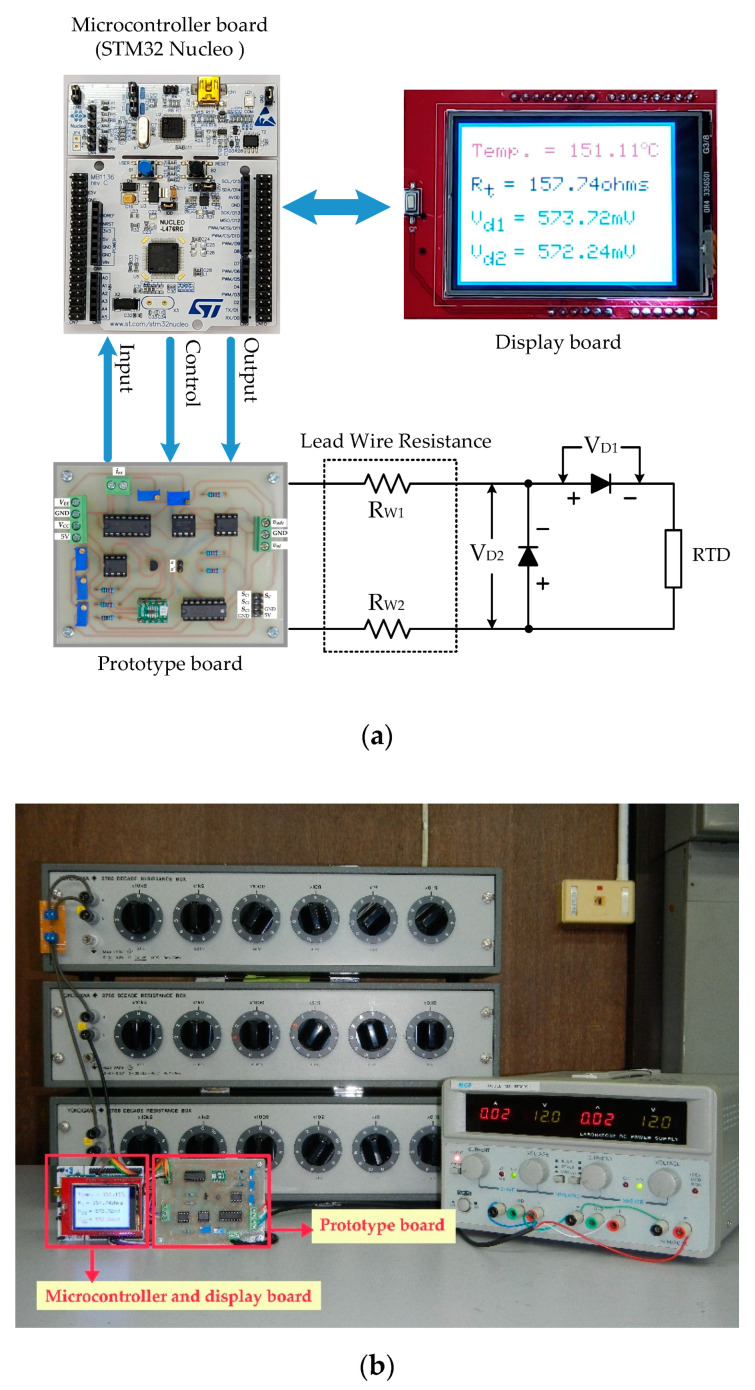
Experimental setup for microcontroller as MCU: (**a**) block diagram; (**b**) experiment prototype.

**Figure 9:**
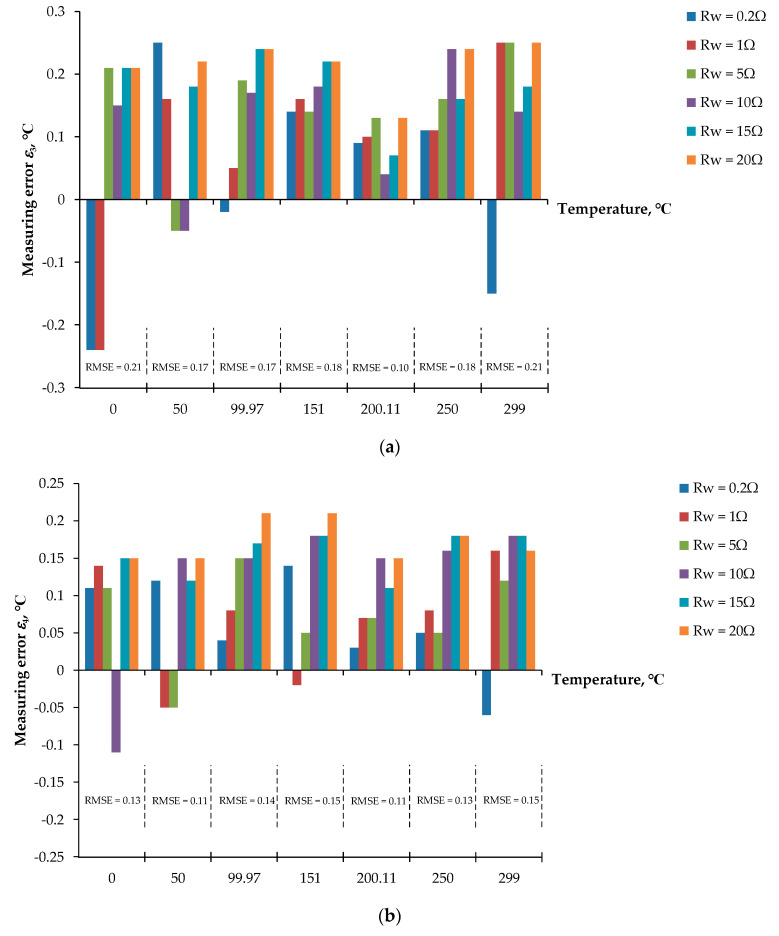
Errors of temperature measurement for MCU using microcontroller: (**a**) temperature error for pt100; (**b**) temperature error for pt1000.

For the second signal processing unit, Equations (5) to (11) were placed on the microcontroller board. The dashed line *a* and *a*´ was connected by the jumper. The analog switches *S_C_*_1_ to *S_C_*_3_ and *S_p_* were governed by the microcontroller board to generate the reference voltage signal *V_ref_*. The voltage signal *V_ref_* was converted to the excitation current *i_ex_* by the voltage-to-current converter, same as the three-step current signal shown in Figure 6c. It should be noted that the ADC was included with the microcontroller board. Therefore, the AIO board was not required for the second experiment. The block diagram for the microcontroller included the display, and the RTD is shown in Figure 8a. The experiment prototype for the second experiment is shown in Figure 8b. The RTD and the length of the lead wire are represented by the resistance decade box. The evaluation of the second experiment is under the same condition as the first experiment. Table 3 and Table 4 show the measured resistances *R_t_* and the diode voltages *V_D_*_1_ and *V_D_*_2_ for pt100 and pt1000, respectively. The errors *ε*_3_ and *ε*_4_ of the measured temperature for pt100 and pt1000 are shown in Figure 9a,b, respectively.

The maximum errors of about 0.25 °C and 0.21 °C for the pt100 and pt1000, respectively, were achieved. The maximum errors of *ε*_3_ and *ε*_4_ were higher than the maximum errors of *ε*_1_ and *ε*_2_, respectively. This is because the resolution of the ADC provided in the Nucleo-64 microcontroller board was less than the AIO board. The RMSE for the dataset of the measured errors *ε*_3_ and *ε*_4_ was in the range of 0.10 °C to 0.21 °C and 0.11 °C to 0.15 °C, respectively. It should be noted that the second experiment is attractive in terms of low cost, small size, and simple configuration.

**Figure 10:**
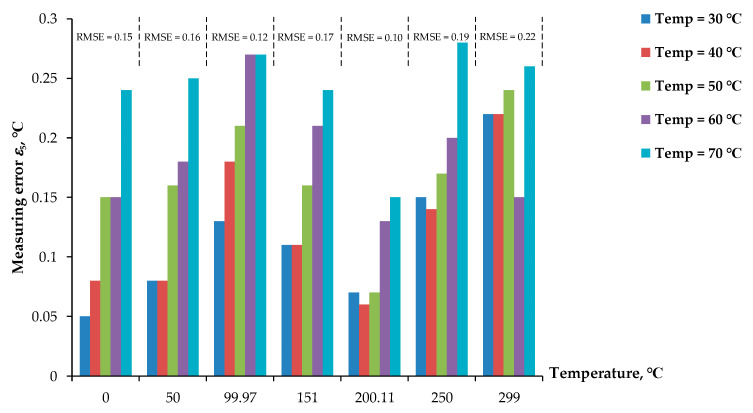
Measurement error *ε*_5_ of pt100 for 30 m lead wire with the ambient temperatures varied from 0 °C to 70 °C.

In addition, the lead wires of the pt100 RTD were extended to 30 m for remote temperature measurement using the Nucleo-64 microcontroller board. The extended lead wire used in this experiment was a 26AWG two-conductor copper wire. The resistance of the extended lead wire and the temperature coefficient were 0.134 Ω/m and 0.0039 Ω/Ω°C, respectively [38]. Therefore, the lead-wire resistance of about 2.04 Ω was calculated [11]. The ambient temperature of the lead wire and the diodes varied from 30 °C to 70 °C. Table 5 shows the measured temperature, resistance *R_t_*, and the diode voltages *V_D_*_1_ and *V_D_*_2_. The measurement error *ε*_5_ of the measured temperature for the variation of the RTD temperature varied from 0 °C to 300 °C, as shown in Figure 10. A maximum error of about 0.27 °C was observed. The RMSE for the dataset of the measured error *ε*_5_ was in the range of 0.10 °C to 0.22 °C.

The first and second experiments show that the proposed procedure can accurately readout the RTD resistance *R_t_* and the diode voltages *V_D_*_1_ and *V_D_*_2_. The proposed technique exhibits the effectiveness of the remote measurement systems using the resistive transducer. Furthermore, this technique can be provided to determine and compensate the wire resistance for the long-distance communication between the control station and the sensor or actuator to acquire a certain signal.

## 5. Conclusions

A procedure for the precise determination of the RTD resistance and the lead-wire resistance was introduced. The technique is based on the use of a three-step current signal to excite the RTD, where the magnitude of each step of the three-step current signal is double that of the previous step. The power dissipation in the RTD used in this experiment was about 3.06 μW for the pt100 RTD, which is very small. Therefore, the self-heating error in the temperature of the RTD can be prevented. The performance of the proposed technique was confirmed by the experimental implementation. The experimental results show that the RTD resistance and the lead-wire resistance can be accurately determined without the requirement of well-matched devices used in the traditional approaches. The maximum error of the temperature measurement from the pt100 RTD of about 0.27 °C was observed when the lead wire was placed in various temperatures of the environment, from 30 °C to 70 °C. The proposed technique using a microcontroller exhibits the advantages in terms of high accuracy, simple configuration, low cost, and compactness. The proposed circuit technique is suitable for two-wire resistive transducers such as the RTD, strain gauge, and resistive displacement transducer, and can be operated in a harsh environment.

## Figures and Tables

**Table 1:** Measured results for RTD (pt100) with different lead-wire resistances using AIO.

Lead-Wire Resistance (Ω) at 30 °C	Temperature (°C) pt100 RTD (Ω)							Diode Voltage (mV) at 30 °C and *I_D_* = 1 mA	
								*V_D_*_1_	*V_D_*_2_
0.00	0.00 100.00	50.00 119.40	99.97 138.50	151.00 157.70	200.11 175.90	250.00 194.10	299.00 211.70	600.68	598.21
0.20	−0.03 99.99	50.05 119.42	99.89 138.47	150.89 157.74	200.14 175.91	250.03 194.11	298.94 211.68	600.68	598.71
1.00	0.08 100.03	50.18 119.47	100.05 138.53	151.16 157.76	200.14 175.91	250.08 194.03	299.03 211.71	600.71	599.28
5.00	0.05 100.02	50.16 119.46	100.10 138.55	151.14 157.75	200.19 175.93	250.08 194.03	299.03 211.71	600.78	599.64
10.00	0.11 100.04	50.13 119.45	100.10 138.55	151.14 157.75	200.19 175.93	250.08 194.03	299.11 211.74	601.04	599.77
15.00	0.08 100.03	50.13 119.45	100.07 138.54	151.16 157.76	200.22 175.94	250.11 194.14	299.11 211.74	600.75	598.25
20.00	0.11 100.04	50.16 119.46	100.13 138.56	151.16 157.76	200.27 175.96	250.11 194.14	299.17 211.76	601.07	599.86

**Table 2:** Measured results for RTD (pt1000) with different lead-wire resistances using AIO.

Lead-Wire Resistance (Ω) at 30 °C	Temperature (°C) pt1000 RTD (Ω)							Diode Voltage (mV) at 30 °C and *I_D_* = 1 mA	
								*V_D_*_1_	*V_D_*_2_
0.00	0.00	50.00	99.97	151.00	200.11	250.00	299.00	600.67	598.19
	1000.00	1194.00	1385.00	1577.00	1759.00	1941.00	2117.00		
0.20	0.05	50.05	100.04	150.92	200.12	249.95	299.14	600.67	598.21
	1000.19	1194.19	1385.27	1576.70	1759.04	1940.82	2117.50		
1.00	0.02	49.94	99.95	150.92	200.14	249.95	299.08	601.44	599.51
	1000.08	1193.77	1384.92	1576.70	1759.07	1940.82	2117.29		
5.00	−0.02	50.06	100.05	151.05	200.14	250.11	299.08	601.41	599.49
	999.92	1194.23	1385.30	1577.19	1759.07	1941.40	2117.29		
10.00	0.05	50.08	100.08	151.05	199.98	250.11	298.93	601.41	599.48
	1000.19	1194.3	1385.42	1577.19	1758.52	1941.4	2116.75		
15.00	−0.02	50.11	100.12	151.10	200.23	250.13	299.12	602.15	600.26
	999.92	1194.42	1385.57	1577.37	1759.44	1941.47	2117.43		
20.00	0.04	50.08	100.12	151.14	200.23	250.15	299.12	601.93	600.11
	1000.02	1194.30	1385.57	1577.52	1759.44	1941.54	2117.43		

**Table 3:** Measured results for RTD (pt100) with different lead-wire resistances using microcontroller board.

Lead-Wire Resistance (Ω) at 30 °C	Temperature (°C) pt100 RTD (Ω)							Diode Voltage (mV) at 30 °C and *I_D_* = 1 mA	
								*V_D_*_1_	*V_D_*_2_
0.00	0.00	50.00	99.97	151.00	200.11	250.00	299.00	600.65	598.20
	100.00	119.40	138.50	157.70	175.90	194.10	211.70		
0.20	−0.24	50.25	99.95	151.14	200.20	250.11	298.85	600.65	598.19
	99.91	119.50	138.48	157.75	175.93	194.57	211.65		
1.00	−0.24	50.16	100.02	151.16	200.21	250.11	299.25	600.67	598.45
	99.91	119.46	138.52	157.76	175.94	194.57	211.79		
5.00	0.21	49.95	100.16	151.14	200.24	250.16	299.25	599.87	598.45
	100.08	119.38	138.57	157.75	175.99	194.16	211.79		
10.00	0.15	49.95	100.14	151.18	200.15	250.24	299.14	599.94	598.86
	100.06	119.38	138.56	157.77	175.95	194.17	211.75		
15.00	0.21	50.18	100.21	151.22	200.18	250.16	299.18	601.12	599.23
	100.08	119.47	138.59	157.78	175.97	194.16	211.76		
20.00	0.21	50.22	100.21	151.22	200.24	250.24	299.25	601.12	599.24
	100.08	119.48	138.59	157.78	175.99	194.17	211.79		

**Table 4:** Measured results for RTD (pt1000) with different lead-wire resistances using microcontroller board.

Lead-Wire Resistance (Ω) at 30 °C	Temperature (°C) pt1000 RTD (Ω)							Diode Voltage (mV) at 30 °C and *I_D_* = 1 mA	
								*V_D_*_1_	*V_D_*_2_
0.00	0.00 1000.00	50.00 1194.00	99.97 1385.00	151.00 1577.00	200.11 1759.00	250.00 1941.00	299.00 2117.00	600.68	598.19
0.20	0.11 1000.42	50.12 1194.46	100.01 1385.15	151.14 1577.52	200.14 1759.11	250.05 1941.18	298.94 2116.78	600.68	598.20
1.00	0.14 1000.53	49.95 1193.81	100.05 1385.3	150.98 1576.93	200.18 1759.26	250.08 1941.29	299.16 2117.58	601.1	598.58
5.00	0.11 1000.42	49.95 1193.81	100.12 1385.57	151.05 1577.19	200.18 1759.26	250.05 1941.18	299.12 2117.43	600.9	599.11
10.00	−0.11 999.58	50.15 1194.57	100.12 1385.57	151.18 1577.67	200.26 1759.56	250.16 1941.58	299.18 2117.65	601.14	599.30
15.00	0.15 1000.57	50.12 1194.46	100.14 1385.65	151.18 1577.67	200.22 1759.41	250.18 1941.65	299.18 2117.65	600.86	599.31
20.00	0.15 1000.57	50.15 1194.57	100.18 1385.8	151.21 1577.78	200.26 1759.56	250.18 1941.65	299.16 2117.58	600.87	599.26

**Table 5:** Measured results for RTD (pt100) for 30-m lead wire with different ambient temperatures using microcontroller board.

Ambient Temperature (°C)	Temperature (°C) pt1000 RTD (Ω)							Diode Voltage (mV) at *I_D_* = 1 mA	
								*V_D_*_1_	*V_D_*_2_
0.00	0.00 100.00	50.00 119.40	99.97 138.50	151.00 157.70	200.11 175.90	250.00 194.10	299.00 211.70	-	-
30.00	0.05 100.20	50.08 119.43	100.10 138.55	151.11 157.74	200.18 175.93	250.15 194.15	299.22 211.78	600.68	598.19
40.00	0.08 100.03	50.08 119.43	100.15 138.57	151.11 157.74	200.17 175.92	250.14 194.15	299.22 211.78	573.72	572.24
50.00	0.15 100.06	50.16 119.46	100.18 138.58	151.16 157.76	200.18 175.93	250.17 194.16	299.24 211.79	544.71	543.55
60.00	0.15 100.06	50.18 119.47	100.24 138.60	151.21 157.78	200.24 175.95	250.20 194.17	299.15 211.75	522.26	521.88
70.00	0.24 100.09	50.25 119.50	100.24 138.60	151.24 157.79	200.26 175.96	250.27 194.20	299.26 211.79	500.37	499.05

## Data Availability

Not applicable.
